# Sprouts Use as Functional Foods. Optimization of Germination of Wheat (*Triticum aestivum* L.), Alfalfa (*Medicago sativa* L.), and Radish (*Raphanus sativus* L.) Seeds Based on Their Nutritional Content Evolution

**DOI:** 10.3390/foods11101460

**Published:** 2022-05-18

**Authors:** Helga Francis, Espérance Debs, Mohamed Koubaa, Zeina Alrayess, Richard G. Maroun, Nicolas Louka

**Affiliations:** 1 Centre d’Analyses et de Recherche, Unité de Recherche Technologies et Valorisation Agro-Alimentaire, Faculté des Sciences, Saint Joseph University of Beirut, Riad El Solh, P.O. Box 17-5208, Beirut 1104 2020, Lebanon; helga.francis@usj.edu.lb (H.F.); rayesszeina@gmail.com (Z.A.); richard.maroun@usj.edu.lb (R.G.M.); nicolas.louka@usj.edu.lb (N.L.); 2 Department of Biology, Faculty of Arts and Sciences, University of Balamand, P.O. Box 100, Tripoli 1300, Lebanon; esperance.debs@balamand.edu.lb; 3 Centre de Recherche Royallieu, ESCOM, TIMR (Integrated Transformations of Renewable Matter), University of Technology of Compiègne, CS 60319, CEDEX, 60203 Compiègne, France

**Keywords:** sprouts, functional food, germination, vitamins, polyphenols

## Abstract

Wheat, alfalfa, and radish sprouts are well-renowned for their high nutritional content. However, their optimal imbibition and germination durations are rarely considered in the literature. In this study, reduced imbibition times of 3 h, 10 h, and 4 h were demonstrated for the wheat, alfalfa, and radish seeds, respectively. The evolution of their crude fat, proteins, polyphenols, antioxidant activity, and vitamins were investigated over 7 days of germination. The crude fat and protein loads of these sprouts slightly varied during germination, whereas the phenolic compounds and antioxidant activity maxed out at day 7, 5, and 6 for the wheat, alfalfa, and radish sprouts, respectively, with significant levels of catechin. The vitamins highly increased, showing noteworthy yet different peaks of growth depending on the seed and the vitamin analyzed. Interestingly, alfalfa and radish sprouts, taken at their optimal germination day, would decidedly contribute to meet our Recommended Daily Allowances (RDAs) of vitamins E, A, and B6. Overall, for a greater nutritional content and a potential use of these sprouts as nutraceutical ingredients, our results suggested to leave the wheat, alfalfa, and radish seeds to germinate only over 7, 4, and 6 days, respectively, after which their nutritional quality tended to decrease.

## 1. Introduction

In a world increasingly seeking an optimal nutrition, germination has gained a major interest, especially for health-conscious consumers. Once germinated, sprouts can be consumed raw, or even cooked since their preservation is brief due to microbial deterioration. These sprouts are known to have a higher nutritional value and a better digestibility than their corresponding seeds [1,2,3]. In fact, germination is the process during which a vegetable seed, a legume, or a cereal grows into a seedling. It generally begins following the imbibition phase where the seed absorbs enough water, and ends with the emergence of the embryonic axis [4,5]. This process is accompanied by major modifications in the nutritional and biochemical characteristics of the seeds. Many preexisting enzymes are activated, whereas others are newly formed; proteins and amino acids are augmented [4,6], and some vitamins are amplified, whereas some others are synthesized [4,7,8].

The germinated seeds are rich in phytochemicals, specifically flavonoids and phenolic acids [9,10], that possess important biological activities, including anti-radical, anti-mutagenic, anti-estrogenic, anti-cancer, and anti-inflammatory effects, which may be beneficial to human health in the prevention of cancer, cardiovascular diseases, and other pathologies [11]. All of these potentials promote the use of germinated seeds nowadays.

The germination of many edible seeds has been frequently tackled in the literature through the passing years. Numerous authors worked on different varieties of wheat [12,13,14,15,16], whereas fewer studied mung beans, buckwheat, broccoli, radish, alfalfa, chickpeas, brown rice, etc. [1,17,18,19,20,21,22,23].

Three species were the subject of our study: wheat, alfalfa, and radish. Wheat is the most widely consumed cereal grain in the world. Being the basic ingredient of bread, it highly contributes to fulfilling our daily nutritional needs in carbohydrates, proteins, minerals, vitamins, and phytocompounds. Producing wheat sprouts with improved nutritional content is, therefore, of prime interest to us. Similarly, the alfalfa seed, more commonly called lucerne, was initially highly valued for animal feed. Then, it became, in its germinated form, an attractive option for humans. It was proven to be rich in vitamins, polyphenols, proteins, and many other nutritive substances [23,24]. Finally, radish is also known for its rich nutritional content in proteins, antioxidants, polyphenols, and vitamins [19,25,26], and for its sprouts that are largely consumed nowadays.

Since domestic practice, as well as the international recommendations, suggest distinct and often differing germination durations for each type of seed, our ultimate aim was to define the optimal duration of germination, i.e., the ideal time to consume or use these sprouts. Consequently, the purpose of this study was to investigate the evolution of the nutritional content of wheat, alfalfa, and radish seeds with germination. Changes in their vitamins (B2, B6, E, and β-carotene), lipids, proteins, polyphenols, and antioxidant activity were closely monitored over 7 days of germination. Unlike most of the previous works, measurements were done for every nutrient on a daily basis, which explains the uniqueness of this study.

## 2. Materials and Methods

### 2.1. Materials

Organic whole soft winter wheat (*Triticum aestivum* L.), alfalfa (*Medicago sativa* cv. *Luzelle*), and Daikon radish (*Raphanus sativus* var. *longipinnatus*) one-year-old seeds were purchased from MARKAL Produits Alimentaires (St. Marcel-lès-Valence, France) via Naturalia s.a.l. (Baabda, Lebanon).

All reagents were of analytical or HPLC grade when necessary. Diethyl ether used for the crude fat analysis was obtained from Scharlau (Barcelona, Spain), whereas all other chemicals, HPLC standards, and solvents were purchased from Sigma-Aldrich Chemical Co. (St. Louis, MO, USA).

In all of our High-Performance Liquid Chromatography (HPLC) analyses, a liquid chromatography (Waters Alliance, Milford, MA, USA) system coupled to a control system and data collection software, Empower 3, was employed.

### 2.2. Germination Process

#### 2.2.1. Germination Conditions

Germination starts with an imbibition (soaking) phase over a duration that varies depending on the study and the type of seeds [19,23,27]. Therefore, the imbibition time was preliminarily optimized in our experiments for each type of seed as described in Section 2.2.2.

Seeds were first soaked in mineral water with a 2:10 (g/mL) mass-to-volume ratio, and mixed every hour for the alfalfa and radish seeds. As for the wheat grains, water was replaced every hour [28]. Water was then removed, and the seeds were spread on a sterilized plastic box covered with humid Whatman No. 1 filter papers. These filter papers were kept wet by regular spraying of mineral water every 12 h. Seeds were left to sprout at 22 °C ± 1 °C for only 7 days, since some microbial growth was observed on the sprouts after this duration.

#### 2.2.2. Determination of the Optimal Imbibition Time for Each Type of Seed

In order to optimize this step, imbibition for several durations (varying from 30 min to 12 h) was performed and the corresponding germination ratios were determined. For this, seeds were soaked in mineral water as described in Section 2.2.1. Afterwards, 50 seeds were left to germinate as described in Section 2.2.1.

The germination ratio was calculated after 24, 36, 48, and 72 h of growth by counting the number of sprouts that developed. The optimal imbibition time was then determined for each type of seed. All experiments were done in quintuplicates.

### 2.3. Drying and Dry Matter Content of Seeds and Sprouts

Samples of sprouts were collected every 24 h, dried at 50 °C in an UFE 700 oven (Memmert GmbH, Schwabach, Germany), ground in a knife mill (IKA M20, Staufen, Germany), and then kept in the dark at −20 °C for further analyses, except for the vitamins analyses. The preliminary study we did on the drying kinetics of the sprouts showed that in order to reach a steady water content where water will not interfere with the results of the analyses, six hours of drying were needed for the alfalfa and radish, and ten hours for the wheat. The dry matter (DM) contents of seeds and sprouts were gravimetrically determined in triplicate after drying approximately 1 g of each sample at 105 °C (WTC Binder oven, Tuttlingen, Germany) for 24 h.

### 2.4. Crude Protein and Fat Contents Determination

Crude proteins were determined according to the Kjeldahl method (AOAC, 1990) with a nitrogen-to-proteins conversion factor of 6.25 for the radish and alfalfa seeds, and 5.7 for the wheat grains. Crude fat contents were measured according to the AOAC method. Analyses were done in triplicates and the results expressed as percentage per dry matter.

### 2.5. Total Soluble Phenolic Content and HPLC Identification and Quantification of the Free Phenolic Acids

#### 2.5.1. Extraction

An amount of 5 g of ground seeds and 3 g of sprouts were extracted with 20 mL of methanol as described by Pająk et al. [19]. The extraction was performed in triplicates.

#### 2.5.2. Total Phenolic Content

The total phenolic contents of dried sprouts were assessed following an adapted Folin–Ciocalteu method. Either 0.1 mL of the extract, a suitable diluted extract, or the different dilutions of the calibration curves were added to 0.4 mL of distilled water and 2.5 mL of 0.2 N Folin–Ciocalteu reagent, and then mixed using a vortex shaker (Zx3, VELP Scientifica, Usmate Velate, Italy). Afterwards, 2 mL of sodium carbonate solution (7.5 g/100 mL) were added. The mixture was then incubated for 10 min at 60 °C, and then 10 min at −20 °C, before measuring its absorbance at 760 nm against a negative control. Data were reported as mean ± standard deviation of three replications and the results expressed as mg of Gallic Acid Equivalent (GAE) per g of DM.

#### 2.5.3. HPLC Identification and Quantification of the Free Phenolic Compounds

In order to identify and quantify their phenolic content, the methanol extracts were analyzed by HPLC. The analytical column used was a Discovery**^®^** HS C18 (5 µ, 250 × 4.6 mm) (Supelco, Bellefonte, PA, USA). The chromatographic separation of 20 µL of extract was performed at 35 °C at a flow rate of 1 mL/min with a gradient elution. The mobile phase consisted of eluent A (2% formic acid in water) and eluent B (69% methanol and 2% formic acid in water). The elution gradient profile was the following: 100% solvent A for the first 3 min, 100% A to 90% in 7 min, 90% to 60% A in 50 min, 60% to 40% A in 20 min, 40% to 20% A in 25 min, 20% to 0% A in 15 min, 0% to 100% A in 20 min. The detection was performed by a scanning between 210 and 600 nm using a UV-Vis Photodiode Array detector (Waters 2998). The standards used were caffeic acid, catechin, chlorogenic acid, cinnamic acid, ferulic acid, gallic acid, p-coumaric acid, p-hydroxybenzoic acid, protocatechuic acid, resveratrol, rutin hydrate, and quercetin.

### 2.6. DPPH Radical Scavenging Activity (RSA)

The determination of the DPPH (2,2-diphenyl-1-picrylhydrazyl) radical scavenging activity of the radish and alfalfa seeds and sprouts was performed on the methanolic extract (obtained in Section 2.5.1), using Trolox (6-hydroxy-2,5,7,8-tetramethylchroman-2-carboxylic acid) as a control. Succinctly, an aliquot of 50 µL of the methanolic extract, its dilution, or the standard (Trolox) were added to 1450 µL of the DPPH solution (2.3 mg DPPH in 100 mL methanol). After 30 min of incubation in the dark at room temperature, the absorbance of the mixture was measured using methanol as blank. The percentage of inhibition was then estimated based on the change in absorbance, and the DPPH radical-scavenging activity was expressed as mg of Trolox equivalents per g of DM of seeds or sprouts.

### 2.7. Vitamin Content Analysis

#### 2.7.1. Extractions of Vitamins

Extractions of vitamins were done in triplicates on fresh undried samples of sprouts collected every 24 h, and ground using a knife mill (IKA M20).

Vitamin E and β-carotene extraction. Extraction of vitamin E and β-carotene starts with a saponification where 5 g of ground sample were refluxed at 75 °C for 35 min, using 20 mL of ethanol and 5 mL of potassium hydroxide (12 N). Vitamins were protected from oxidation by Nitrogen flushing and ascorbic acid addition (25 mg). The mixture was then cooled in an ice bath after adding 15 mL of sodium chloride (10 g/L). The unsaponifiable fraction, which includes β-carotene and tocopherols, was then isolated by liquid-liquid extraction using 2 × 10 mL of hexane:ethyl acetate (9:1, *v*/*v*). Afterwards, the solvent was evaporated under vacuum, and the residue reconstituted in 5 mL of methanol:tetrahydrofurane (95:5, *v*/*v*). Vitamin extracts were finally filtered through a PTFE membrane (0.45 µm).

Vitamin B2 and B6 extraction. For the simultaneous extraction of these vitamins from seeds and sprouts, both acidic and enzymatic hydrolyses are usually recommended [29,30]. To this end, 10 mL of hydrochloric acid (0.1 M) were added to 3 g of ground sample. The pH of the mixture was then adjusted to 4.5 using sodium acetate (1 M). An amount of 40 mg of papain and α-amylase was added, and the mixture was incubated at 37 °C for 18 h. Samples were then centrifuged, and extracts were isolated and filtered.

#### 2.7.2. Assay of Vitamins by HPLC

HPLC analyses of vitamins were carried out on a Discovery**^®^** C18 (5 µ, 250 × 4.6 mm) (Supelco, Bellefonte, PA, USA). The vitamin E analysis was performed by isocratic elution at 30 °C and a flow rate of 1 mL/min. The mobile phase consisted of methanol:water (95:5, *v*/*v*). The detector was set to wavelengths of excitation of 396 nm/emission 340 nm, and the injection volume was 20 µL of the sample extract. The standard used was the α-tocopherol. For the β-carotene quantification, an isocratic solvent system consisting of dichloromethane:acetonitrile:methanol (20:70:10, *v*/*v*/*v*) at a flow rate of 1.0 mL/min was used [31]. The column temperature was set at 35 °C and the detection was performed at 445 nm with a UV-Visible detector using β-carotene as a standard. The vitamin A activity was estimated based on the following formula published by the U.S. Department of Agriculture (USDA): 1 retinol activity equivalent (μg RAE) = 12 μg of dietary β-carotene. Vitamins B2 and B6 analysis was performed in a single run of 60 min using gradient elution at a constant flow rate of 1 mL/min. The solvent system consisted of eluent A (50 mM K**_2_**HPO**_4_**, pH 7) and eluent B (methanol). The elution was programmed as follows: 0–7 min isocratic 99% A, then a linear decrease from 99% A to 70% A in 25 min, then isocratic 70% A for 7 min, followed by a linear increase from 70% A to 99% A in 10 min, and finally isocratic 99% A for the last 11 min. The injection volume was of 30 µL and the column temperature was set at 35 °C. The fluorescence detector was set to wavelengths of excitation of 450 nm/emission 530 nm for the vitamin B2, and excitation 290 nm/ emission 390 nm for the vitamin B6. Quantification was achieved using the riboflavin (B2) and pyridoxine hydrochloride (B6) standards.

## 3. Results

### 3.1. Determination of the Optimal Imbibition Time for Each Type of Seed

Studying the germination rate enabled us to define an ideal imbibition time for each type of seed. Indeed, wheat grains reached a rate of 100% of germination starting at 3 h of imbibition; shorter imbibition times generated lower rates of germination. For the alfalfa seeds, a 10 h imbibition was adopted, leading to a maximum germination rate of 88%, whereas radish needed 4 h of soaking to reach a maximum germination rate of 84%. Seeds of alfalfa and radish, soaked for more than 10 and 4 h, respectively, directly reached their highest germination rates after 24 h of germination, but these rates were still lower than the maxima reached at the chosen optimal imbibition duration. On the other hand, seeds soaked for less than 10 and 4 h needed a longer germination duration to reach their highest germination rates, which increased the risk of microbial growth. Our results put forward reduced imbibition time lengths compared to the ones recorded in the literature for the wheat and alfalfa seeds where they varied between 6 h [28], 12 h [19,32], 17 h [33], and 24 h [34,35,36,37]. They also accounted for the unexplained 4 hours’ imbibition reported by Xiao et al. [38] for the radish seeds, which are usually soaked for a minimum duration of 5 or 6 h [39,40].

**Figure 1:**
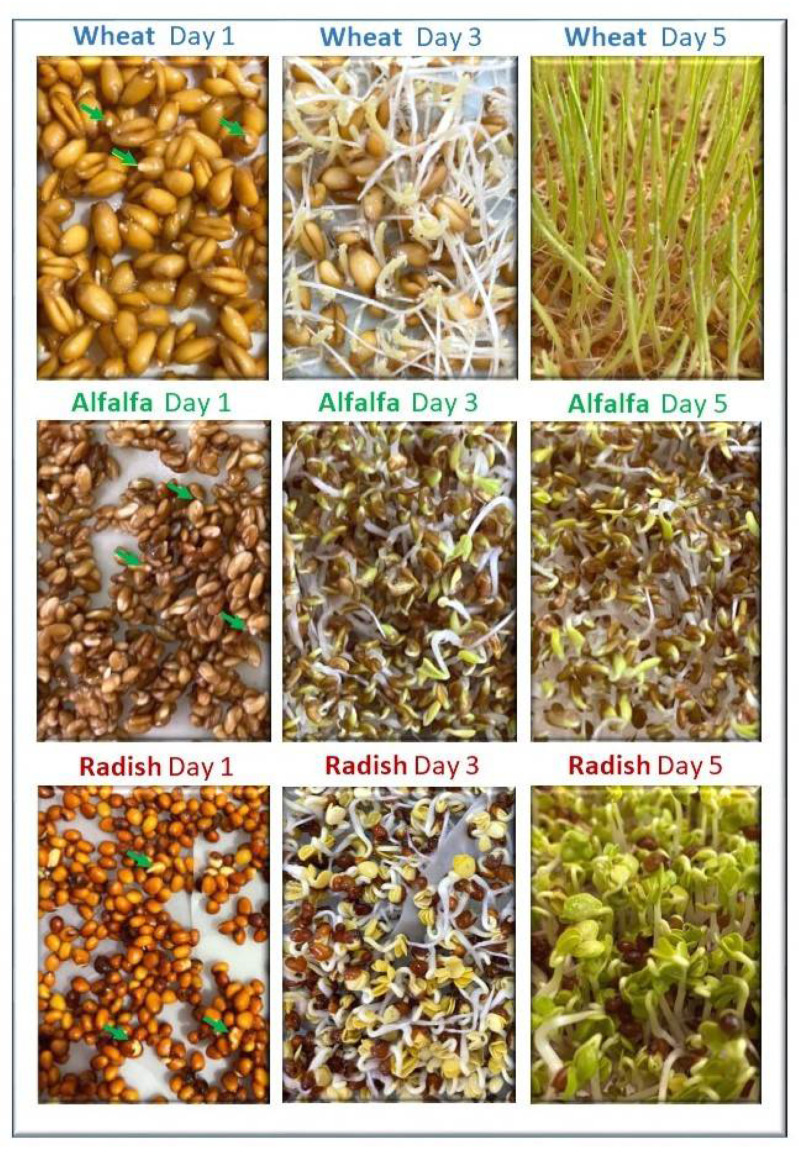
Germination of wheat, alfalfa, and radish sprouts. Green arrows on day 1 indicate the beginning of germination.

Based on these results, seeds were soaked and left to germinate for 7 days. Samples of sprouts were collected every 24 h and their nutritional content was analyzed in order to define the optimal germination duration for each type of seed. Figure 1 represents the development of wheat, alfalfa, and radish sprouts at day 1, 3, and 5. The start of germination is indicated by the green arrows on the day 1 samples.

### 3.2. Crude Fat and Protein Content Evolution with Germination

As has been generally documented, germination triggers several enzymatic reactions leading to the breakdown of proteins and lipids [4,6,10]. In our study, the total crude fat of alfalfa and radish seeds significantly diminished starting at day 3 for the alfalfa, and day 5 for the radish, with a total reduction of 40.4% and 27.6%, respectively, after 7 days of germination. According to previous studies, the fat content of such seeds was stated to either remain unaffected or decrease with germination [36,41,42]. For the alfalfa seeds, Huang and Grunwald [43] reported a 35% diminution in total lipids after 4 days of germination, going from 118 mg/g at day 0 to 77 mg/g at day 4, whereas Marton et al. [36] observed a decrease of 56% after 7 days, going from 10.3 to 4.5% at day 7 with a lipid content of 9.8% at day 3, i.e., a decrease of 4.8% in 3 days. As for the radish sprouts, their lipid content percentages were 39% at day 0, 39.2% at day 2, and 20.2% at day 6 of germination, i.e., a decrease of approximately 48% in 6 days [36]. Compared with the literature, our experiments showed lower percentages of decrease, which may result in slightly higher content in fat-soluble compounds in the final product. In contrast, the total fat of our wheat sprouts remained unchanged until day 3, then showed a 52.5% increase starting at day 0 as presented in Table 1. Singkhornart and Ryu [28] revealed a 25% decrease in wheat, whereas other authors reported an unchanged crude fat content after 3 days and 5 days of sprouting [36,41]. Although storage lipids are metabolized to supply the energy necessary for the germination, structural lipids may increase, hence, reflecting the newly membranes formed [43,44]. This might have influenced our results for the wheat sprouts suggesting distinct metabolic pathways when compared to alfalfa and radish sprouts.

As for the protein content, our experiments showed that 7 days of germination led to a total growth of the crude proteins content of 27.1% for wheat grains, 22.3% for alfalfa, and only 12.7% for radish, occurring on day 5, 2, and 3, respectively. With the exception of a few studies [42] which observed no significant changes in the protein content after 48 h of sprouting for the wheat seeds, the literature data suggested an increase of 5 to 10% in this regard [4,10,41,45]. Our results corroborated this general tendency, although, to our knowledge, no previous data were recorded for alfalfa nor radish sprouts. The launching of metabolic machinery, especially the anabolic one pertaining to growth of the seedling, could be the reason behind the observed increase in protein content.

### 3.3. Total Soluble Phenolic Content Evolution during Germination (TPC)

Our results matched previous studies confirming that sprouts had higher phenolic contents compared to their corresponding seeds [13,14,15,17,22,23,33,41]. However, it was not clear how the level of phenolics varied throughout the germination process. Therefore, and in order to grow edible sprouts with enhanced nutraceutical properties, it was important to study the evolution of the phenolic content during sprouting, and identify their optimal level.

**Figure 2:**
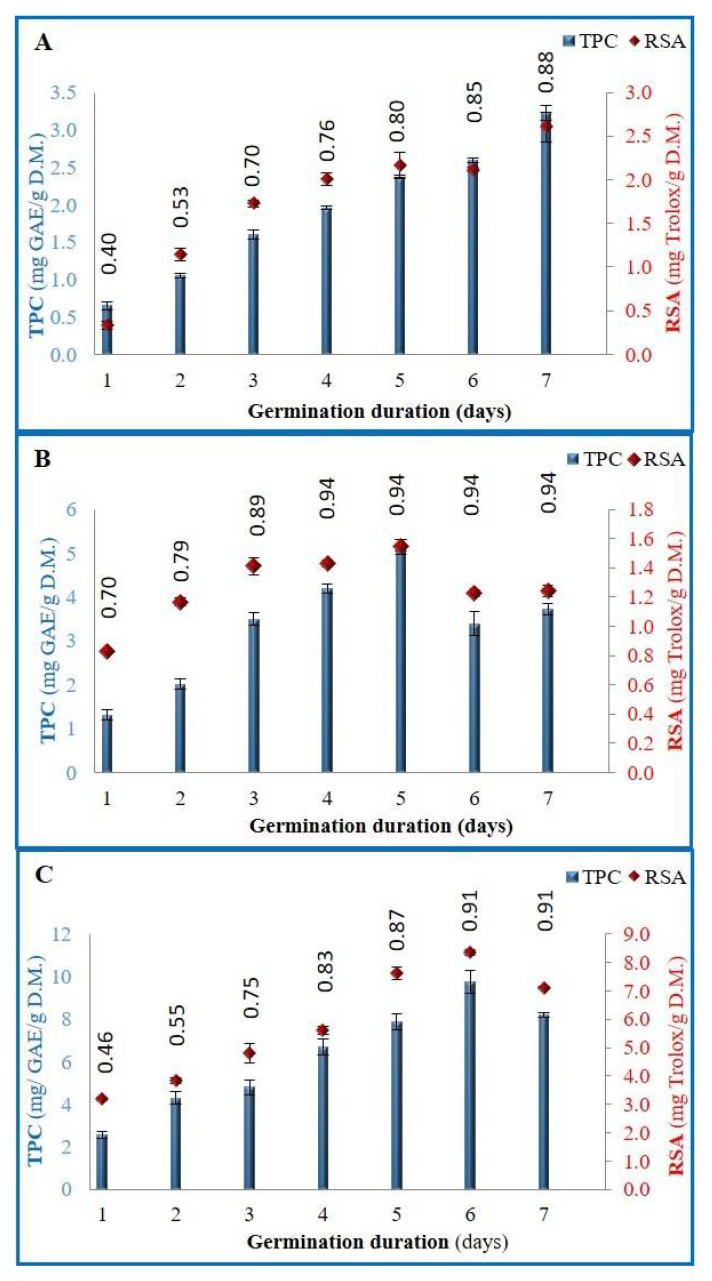
Total phenolic compounds (TPC) and radical scavenging activity against DPPH (RSA) of wheat (**A**), alfalfa (**B**), and radish (**C**) sprouts. Numbers on the graph represent the water content average of the sprouts at the corresponding day.

Concentrations of TPC in wheat sprouts continuously grew with germination, from day 1 to day 7 by 456.3% (Figure 2). On the other hand, TPC in alfalfa sprouts improved gradually from day 1 to day 5, with a rise of 295.4%, and then decreased at days 6 and 7 (−34.5%). As for the radish sprouts, TPC went from 2.56 to 9.75 mg GAE/g DM on day 6 (+280.9%), and then decreased to 8.20 mg GAE/g DM (−15.9%) at day 7. This enrichment in the polyphenolic amount was also observed by Cevallos-Casals and Cisneros-Zevallos [33], who reported an increase of 409% and 535% in phenolic content on a dry matter basis from dormant seeds to 7-day-sprouts for the alfalfa and wheat seeds, respectively. As for the radish seeds, they detected a 63% increase after 7 days of germination, whereas Pająk et al. [19] reported a 206% rise after 5 days of germination. Moreover, Tarasevičienė et al. [46] reported a total phenolic content increase in radish and alfalfa sprouts after 5 days of germination of 370% and 120%, respectively.

A complex biochemical metabolism taking place during germination leads to important changes in the phenolic composition of the seeds [8,47]. These changes indicate the seeds’ preparation towards adverse environments [4,33], such as the microbial growth induced by the high water activity in the sprouts.

It is noteworthy that our results showed that the evolution of the phenolic content and the antioxidant activity is concomitant with that of the water content (Figure 2). As a matter of fact, the water content of the wheat seeds followed the same trend of growth as the TPC and RSA during the 7 days of germination. In the case of alfalfa and radish seeds, it gradually grew and reached its maxima starting at day 4 and 6, respectively. Referring to this observation, the rise in the phenolic content and the antioxidant activity may have manifested as a defensive reaction of the sprouts against the potential microbial growth during germination; the sprouts with high water and nutrients content being a better environment for the proliferation of microorganisms.

### 3.4. DPPH Radical Scavenging Activity (RSA)

As shown in Figure 2, RSA followed the same general trend as the total phenolic compounds for all of the studied sprouts. Wheat sprouts demonstrated a continuous growth of 673.9% between day 1 and day 7, whereas alfalfa and radish sprouts showed a peak of growth at day 5 of 61.8% and day 6 of 515.6%, respectively. This intensification is mainly due to an increase in the antioxidant content, such as polyphenols and vitamins [4,19]. Tarasevičienė et al. [46] mentioned a significant positive correlation between antioxidant activity and phenolic compounds of wheat, radish, and alfalfa sprouts.

Our experiments were validated by the data found in the literature. Indeed, antiradical activity rose with germination for all of the studied seeds [19,33,48]. For the wheat and alfalfa sprouts, Cevallos-Casals and Cisneros-Zevallos [33] observed an increase in the antioxidant activity of 433% for the wheat and 943% for the alfalfa between the dormant seeds and the 7-day-sprouts. As for the radish sprouts, growth rates of 200% [19] and 117% [33] were reported.

Moreover, our results certified that the optimal durations of germination for these seeds, based on both the antioxidant content and the TPC, is 7 days for the wheat (beyond which, microbial development occurred), 5 days for the alfalfa, and 6 days for the radish seeds. Among the tested seeds, radish showed the highest phenolic content (9.75 mg GAE/g DM at day 6), followed by the alfalfa (5.18 mg GAE/g DM at day 5), then the wheat seeds (3.2 mg GAE/g DM at day 7). Moreover, radish sprouts had the most important radical scavenging activity. Interestingly, wheat had higher RSA compared to the alfalfa, even though its phenolic compounds were lower. Studying the antioxidant activity of the sprouts per unit of polyphenols allowed us to evaluate the quality of the polyphenolic compounds of each type of seed.

**Figure 3:**
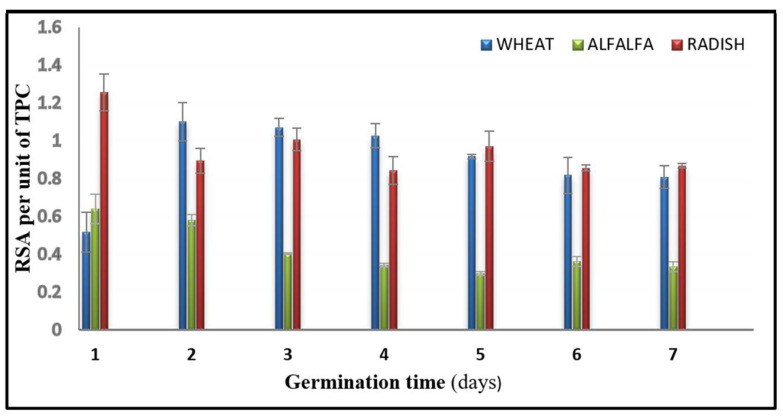
Radical scavenging activity per unit of polyphenols over 7 days of germination for the wheat, alfalfa, and radish sprouts.

Figure 3 shows the evolution of the antioxidant activity per unit of polyphenols (1 mg) over the 7 days of germination for the tested seeds. The highest RSA per unit for the alfalfa and radish seeds appeared on the first day of germination, and then it started to decrease. Wheat polyphenols needed 2 days to reach the highest activity per unit, and then slowly decreased with germination. Wheat seedlings, with the lowest total phenolic compounds among the studied seeds, showed higher RSA activity per unit of TPC compared to the alfalfa and radish sprouts. Overall, the RSA per unit of polyphenols tended to decrease with germination, even though the total phenolic and antioxidant activity of the sprouts increased over the days. This noticeable dissociation between RSA and TPC clearly pinpoints the significance that should be attributed to the quality of TPC over the quantity.

### 3.5. HPLC Identification and Quantification of Some Phenolic Compounds

Phenolic compounds identified in our study for wheat sprouts were comparable to the ones available in the literature [12,15,48]. In fact, our analyses showed that the amounts of caffeic, protocatechuic, p-coumaric, ferulic acids, and catechin increased in the wheat sprouts after 7 days of germination, reaching 9.57, 13.87, 6.91, 14.39 µg/g DM, and 1.44 mg/g DM, respectively. As for the alfalfa sprouts, only catechin and p-coumaric acid were detected, with maxima of 2.95 mg/g DM at day 5 and 5.72 µg/g DM at day 7, respectively. On the other hand, ferulic acid and catechin were detected in the extracts of the radish seeds and sprouts we analyzed, with catechin being the most abundant compound. Their evolution with germination followed the same trend as the total soluble phenolic compounds, with maxima at day 6 of germination for both compounds (3.42 mg/g DM for catechin, and 19.64 µg/g for ferulic acid). Nevertheless, Pająk et al. [19] reported the presence of gallic, protocatechuic, caffeic, p-coumaric, ferulic, chlorogenic, and sinapinic acids in radish seeds and sprouts. The divergence noticed in the phenolic profile and content may be related to several factors, such as plant species and varieties, storage environment, germination conditions, or even the methodology of analysis.

Moreover, the high level of catechin found seems of great interest. The germination induced an important increase in this compound concentration: it grew from 0.13 at day 1 to 2.42 mg/g DM at day 6 for the radish sprouts (i.e., 1761% growth), and from 0.06 to 2.95 mg/g DM at day 5 in the case of the alfalfa sprouts (i.e., 4817% growth). Catechin was reported to have chemoprotective and cardioprotective effects along with antimicrobial properties, proving its health benefits in preventing cancer and heart diseases [49,50,51,52]. It was frequently found and investigated in green tea [51,52], but was also assayed in several grains and sprouts, such as wheat, barley, rye, and oat [41,53]. It will be interesting to further investigate it in the studied seeds and sprouts.

### 3.6. Vitamins Content Evolution with Germination

Sprouts are commonly acknowledged for their high content in vitamins in comparison to unsprouted seeds; although, little is known about the actual evolution of these vitamins during germination. Only Yang et al. [54] studied the kinetics of vitamin E and β-carotene over 9 days of germination for the wheat grains, whereas other authors just evaluated the vitamin contents in unsprouted seeds and sprouts at specific days of germination [30,55,56,57]. To our knowledge, limited data were published about the evolution with germination of the vitamins content of radish and alfalfa sprouts [46,55].

**Figure 4:**
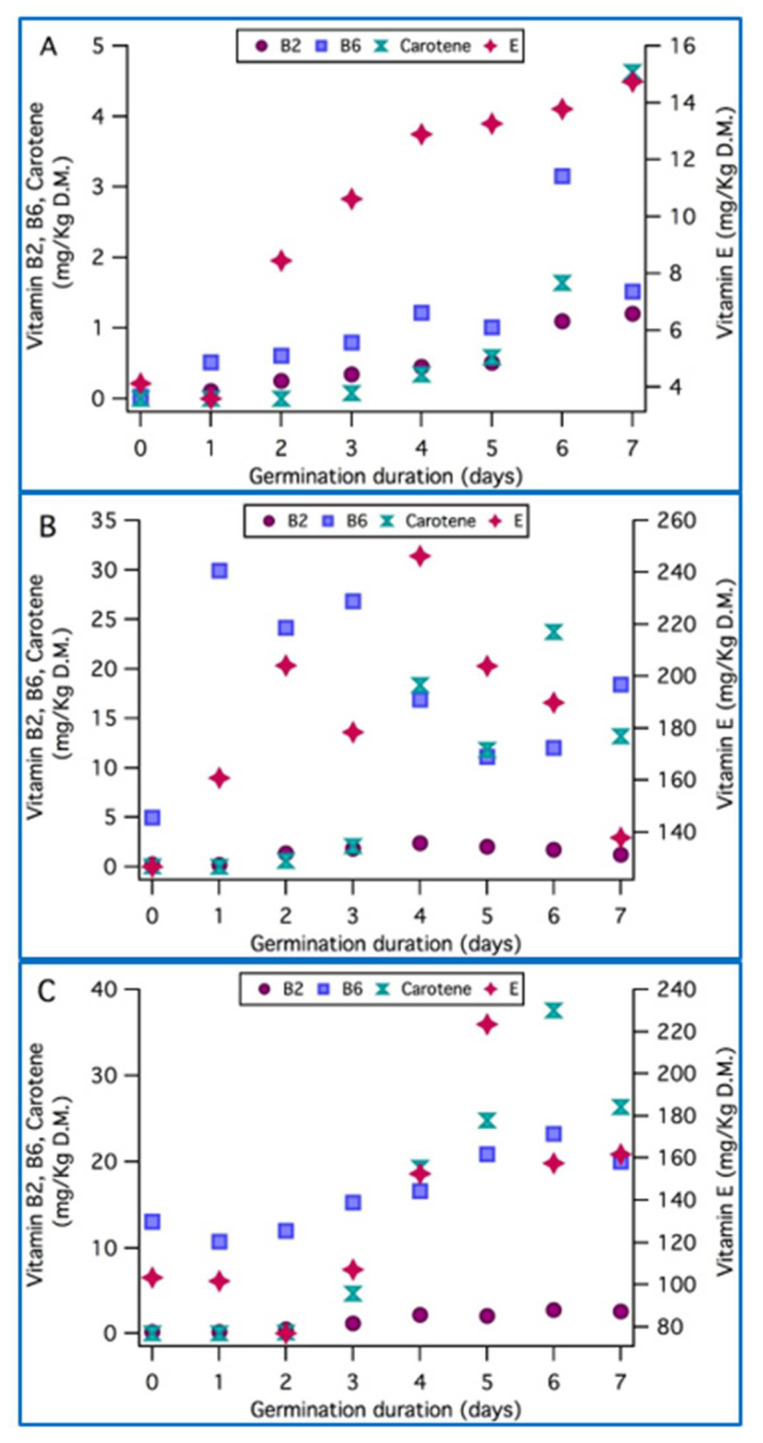
Vitamin B2, vitamin B6, vitamin E, and β-carotene kinetics throughout germination of wheat (**A**), alfalfa (**B**), and radish (**C**) seeds and sprouts, expressed as mg/g DM of seeds.

Figure 4 illustrates the vitamins evolution with sprouting of the three studied seeds. Vitamins of the wheat seeds and sprouts were increasingly changing from day 0 to day 7, with a remarkable elevation of 9501%, 256%, and 5890% for the riboflavin, α-tocopherol, and β-carotene, respectively, except for vitamin B6, which peaked at day 6, with a noteworthy increase of 12,470%, followed by a slight decrease at day 7. Although Plaza et al. [30] noticed a 48% cut in vitamin E in wheat after 4 days of sprouting, other works confirmed the increase of this vitamin with germination [54,56,57]. In the alfalfa sprouts, riboflavin and vitamin E showed peaks of growth at day 4 (800% and 94.5%, respectively), whereas β-carotene needed 6 days of sprouting to reach its maximum (57,700% growth), and then they decreased gradually. Vitamin B6 showed a different response to germination: the highest content being detected at day 1 of sprouting, followed by a fluctuant reduction as shown in Figure 4. As for the radish sprouts, germination led to an overall growth of vitamins, with peaks at day 6 for vitamins B2, B6, and β-carotene, and day 5 for vitamin E, with an improvement of 1083% for vitamin B2, 67% for vitamin B6, 42,438% for β-carotene, and 116% for vitamin E. Zieliński et al. [55] stated that the riboflavin content of 4-day-sprouted radish to be three times higher than that of the original seeds. On the other hand, Tarasevičienė et al. [46] showed that after germination for 5 days, the α-tocopherol contents in wheat, radish, and alfalfa sprouts increased by 5.3 times, 5.4 times, and 6.9 times, respectively. The increasing amounts of vitamins can be associated with the higher metabolic activity taking place in the seedlings.

Radish and alfalfa sprouts showed remarkably higher levels of vitamins than wheat shoots, especially for vitamin E. α-tocopherol levels reached 246.2 mg/kg and 223.5 mg/kg in the radish and alfalfa sprouts, respectively, followed by the wheat sprouts (14.7 mg/kg). Accordingly, it is interesting to consider the consumption of radish and alfalfa sprouts, since their vitamin E content is closely comparable to that of almonds (240 mg/kg according to the USDA), one of the richest sources of α-tocopherol.

In general, an optimal vitamin content will be reached after 7, 6, and 4 days of germination for the wheat, radish, and alfalfa seeds, respectively. Contrasting the vitamins contents of sprouts at their respective optimal day of germination (7 for wheat, 4 for the alfalfa, and 6 for radish) to the Recommended Dietary Allowances (RDAs) of vitamins as published by the USDA underlines the importance of including these seedlings in our daily eating habits. They can provide us with a considerable dose of vitamins, as shown in Table 2. For instance, a cup (33 g) of alfalfa sprouts, consumed at their optimum germination, can fulfill 54% of the recommended daily allowance of vitamin E. Radish sprouts, which are also rich in vitamin E, contain 5.31 mg of tocopherol per cup if consumed at the chosen ideal day of germination (day 4), and its contents can reach 7.37 mg per cup if sprouting is terminated at day 5. Similarly, ingesting a cup of sprouts at their optimum can largely contribute to our RDAs of vitamin A and B6, especially for the radish and alfalfa seedlings.

## 4. Conclusions

Germination, known to alter the nutritional composition of a seed, was proven to decrease its crude fat material, to slightly increase its protein content, and to highly raise its vitamins content (especially vitamin A and E), total phenolic compounds, and antioxidant activity. The improvement in the vitamins content and antioxidant activity, in addition to the increase in catechin found in these sprouts, are encouraging due to their nutraceutical applications. Monitoring the different variables allowed us to establish an optimal duration of germination for each seed where the nutritional content is ideal for consumption and use: 7 days for wheat, 4 days for alfalfa, and 6 days for radish seeds.

Overall, the higher content in polyphenolic acids and flavonoids of these sprouts compared to the unsprouted seeds introduces them as functional food ingredients, and highlights, once again, the added value of integrating them in our daily nutrition for better and healthier food choices.

As a perspective, an innovative drying–texturizing process developed in our laboratories will be applied to dehydrate the germinated seeds picked at their optimal nutritional content. This technique, called “Intensification of Vaporization by Decompression to the Vacuum” (IVDV) [58,59,60], will enable us to preserve the sprouts with a minimal loss of their nutritional value compared to the commonly used drying techniques. The dried sprouts, a valuable source of natural and healthy nutrients, viewed as potential functional food ingredients, will have new applications and a longer shelf life.

## Figures and Tables

**Table 1:** Crude fat and proteins evolution during 7 days of germination for the wheat, alfalfa, and radish seeds and sprouts, determined as percentages (%) per dry matter (DM). Values followed by the same letter within the same column are not significantly different according to Student’s *t* test (*p* = 5%).

	Wheat		Alfalfa		Radish	
Days of Germination	Crude Fat	Crude Proteins	Crude Fat	Crude Proteins	Crude Fat	Crude Proteins
0	2.55 ± 0.01 ^a^	10.42 ± 0.17 ^a^	10.18 ± 0.05 ^a^	36.59 ± 0.20 ^a^	35.11 ± 0.43 ^a^	31.12 ± 0.08 ^a^
1	2.52 ± 0.05 ^a^	10.29 ± 0.17 ^a^	10.22 ± 0.25 ^a^	36.55 ± 0.20 ^a^	35.30 ± 0.43 ^a^	31.06 ± 0.03 ^a^
2	2.54 ± 0.07 ^a^	10.23 ± 0.17 ^a^	10.35 ± 0.21 ^a^	38.36 ± 0.32 ^b^	35.16 ± 0.50 ^a^	31.00 ± 0.04 ^a^
3	2.75 ± 0.03 ^a^	10.67 ± 0.06 ^a^	8.62 ± 0.33 ^b^	39.94 ± 0.37 ^c^	34.67 ± 0.25 ^a^	31.69 ± 0.18 ^b^
4	2.86 ± 0.02 ^b^	10.76 ± 0.12 ^a^	8.49 ± 0.05 ^b^	41.46 ± 0.09 ^d^	35.61 ± 0.46 ^a^	32.04 ± 0.09 ^b^
5	3.01 ± 0.03 ^c^	11.29 ± 0.08 ^b^	7.71 ± 0.13 ^c^	42.86 ± 0.24 ^e^	33.95 ± 0.48 ^b^	33.07 ± 0.02 ^c^
6	3.39 ± 0.03 ^d^	12.20 ± 0.05 ^c^	6.63 ± 0.20 ^d^	43.90 ± 0.21 ^f^	27.49 ± 0.37 ^c^	33.98 ± 0.15 ^d^
7	3.89 ± 0.02 ^e^	13.24 ± 0.13 ^d^	6.07 ± 0.36 ^d^	44.77 ± 0.16 ^g^	25.43 ± 0.18 ^d^	35.07 ± 0.10 ^e^

**Table 2:** Vitamins content of a cup of sprouts, taken at their optimum day of germination, compared to RDAs for women and men between 18 and 50 years old.

		RDAs per Day		Content per Cup (33 g)		
		Women	Men	Wheat	Alfalfa	Radish
Optimal day of germination				Day 7	Day 6	Day 4
Vitamin B2	(mg)	1.3	1.1	0.04	0.08	0.09
Vitamin B6	(mg)	1.3	1.3	0.05	0.56	0.80
Vitamin E	(mg)	15	15	0.49	8.12	5.31
Vitamin A	(µg RAE)	700	900	12.73	50.53	72.44
